# Foot and mouth disease biosecurity and risk mitigation in auction markets: a scoping review

**DOI:** 10.3389/fvets.2025.1659240

**Published:** 2026-01-12

**Authors:** Christy J. Hanthorn, Michael W. Sanderson

**Affiliations:** Department of Diagnostic Medicine and Pathobiology, Center for Outcomes Research and Epidemiology, College of Veterinary Medicine, Kansas State University, Manhattan, KS, United States

**Keywords:** foot and mouth disease, FMD, biosecurity, auction market, scoping review

## Abstract

**Introduction:**

Foot and mouth disease (FMD) is a highly contagious, viral vesicular disease affecting cloven-hoofed livestock species. The disease has negative impacts on livestock health and wellbeing as well as international trade opportunities for affected countries. The United States (U. S.), while currently free from FMD, has developed preparedness and response resources, including biosecurity guidance, for many livestock industry sectors with the aim of balancing robust disease control with business continuity. However, guidance on effective biosecurity strategies for auction markets is lacking.

**Methods:**

Therefore, the objective of this scoping review is to identify peer-reviewed literature that describes biosecurity strategies which decrease the risk of FMD introduction into auction markets or mitigation strategies that prevent FMD transmission from auction markets.

**Results:**

Eight reports were included in this review with six reports discussing FMD biosecurity risks from auction markets, and two reports discussing FMD outbreak mitigation strategies involving auction markets. No reports describing FMD biosecurity risks to or strategies for auction markets were identified for inclusion in this review.

**Discussion:**

This review demonstrates the association between livestock movement through auction markets and FMD introduction risk to herds as well as the potential for auction markets to contribute to widespread dissemination of FMD in the absence of biosecurity strategies. Developing resources to address this current preparedness gap is a critical need illustrated by the evidence identified in this review. Effective biosecurity strategies and risk mitigation guidance for auction markets would substantially bolster the U. S. livestock industry’s resiliency in the event of an FMD incursion

## 1 Introduction

Foot and mouth disease (FMD) is a highly contagious viral disease that often manifests as vesicular lesions and erosions in the mouth and on the feet and teats of affected cloven-hoofed livestock species (1, 2). In addition to the adverse animal health and wellbeing impacts, international trade restrictions are also placed on countries where the disease is present. While the United States (U. S.) is currently free from FMD, the threat of re-introduction has necessitated the development of an array of preparedness and response resources intended to bolster response capabilities in the event of an incursion (3). If FMD were to enter the U. S., efforts to contain and eradicate the disease would likely involve animal movement controls and the required implementation of biosecurity plans (4–7).

Robust disease control efforts will be necessary to eradicate the disease; however, some degree of business continuity for the U. S. livestock industries and their associated marketing avenues must be protected to ensure the survival of the affected industries. In the U. S. auction markets are a primary marketing avenue for many classes of beef cattle, cull dairy cattle, and sheep (8–10), and are utilized to a lesser extent for marketing goats and swine (11, 12). Ideally during an outbreak, auction markets could implement practices to mitigate risk and be allowed to continue operating in a manner that does not amplify disease spread, which will in turn support maintenance of business continuity for several U. S. livestock industries. However, a current lack of biosecurity and risk mitigation guidance for auction markets increases the likelihood that they could serve as a source of a super spreader event through the congregation and dispersal of livestock. This risk makes regulatory officials reluctant to allow their continued operation during a disease outbreak (13).

Biosecurity guidance for foreign animal disease threats has been developed for other sectors of the U. S. livestock industry, including Secure Food Supply (SFS) plans for several livestock species and biosecurity guidance for packing plants (14, 15). The SFS plans recommend enhanced biosecurity practices for premises that maintain groups of livestock in order to keep the herds or flocks from becoming infected; whereas, the biosecurity guidance for packing plants focuses on structural and operational biosecurity measures for the packing plant facility. While some of the principles of existing SFS plans and packing plant guidance are applicable to auction markets, the unique nature of U. S. auction market operations and their business model requires a substantially different approach to biosecurity. Auction markets do not maintain a population of animals, but import new animals from numerous premises for each sale day, sort and recombine them into sale lots, and disperse them to multiple new premises. This uniquely positions auction markets where equal consideration for decreasing disease transmission risk needs to be placed on both the regularly imported animals and the potential for persistence of virus in the physical facility. Biosecurity and risk mitigation strategies specific to the business model of U. S. auction markets are needed to allow them to move livestock through their facilities in a manner that poses a low risk for the importation of infected animals, production of new infections, and the facility becoming contaminated. Allowing ongoing auction market activity without proper risk assessment and disease control efforts could substantially extend an outbreak and result in more harm to the affected livestock industries. However, evaluating the effectiveness of biosecurity and mitigation strategies of foreign animal diseases like FMD in field settings is challenging. Few opportunities exist to evaluate biosecurity or mitigation strategies in auction market settings that operate similarly to U. S. auction markets either in FMD-endemic areas or during FMD outbreaks. Still, reports from the scientific literature may provide information that can be used to inform the development of biosecurity and business continuity strategies for U. S. auction markets in the event of an FMD outbreak. Therefore, the objective of this scoping review is to identify peer-reviewed literature that describes biosecurity strategies which decrease the risk of FMD introduction into auction markets or mitigation strategies that prevent FMD transmission from auction markets. To accomplish this objective, the following questions were developed to guide the review.

1. What is known from peer-reviewed literature about FMD introduction into or transmission within or from an auction market?
2. What is known from peer-reviewed literature about biosecurity strategies (prevention or mitigation) for FMD in an auction market setting?

## 2 Materials and methods

This scoping review was conducted in accordance with the Preferred Reporting Items for Systematic reviews and Meta-Analyses extension for Scoping Reviews (PRISMA-ScR) (16). The objective and review questions were developed *a priori* by group discussion. A protocol for this review was not registered.

### 2.1 Information sources and search strategy

To identify potentially relevant reports, reviewers searched the following bibliographic databases on March 18, 2024 through the Kansas State University library in line with findings from Grindlay et al. (17): CABI Digital library (includes CAB Abstracts and Global Health), Scopus, and Web of Science (includes the Korean Journal Database, Medline, the Russian Science Citation Index, the SciELO Citation Index, the Preprint Citation Index, ProQuest Dissertations and Theses Citation Index, and the Web of Science Core Collection). The dates of coverage, individual search strategies for each database, and number of records returned are described in Table 1. Search terms were drafted by the reviewers (CH, MS) and refined using pilot searches. The final search strategies targeted reports published in English on auction markets or analogous terms (e.g., sale barn or saleyard) AND foot and mouth disease (FMD) AND at least one of our topics of interest (disease introduction, transmission, prevention, mitigation, biosecurity, or cleaning and disinfection strategies). No publication date restrictions were placed on search results. The database search was not supplemented with additional methods to identify sources of evidence.

**Table 1:** Databases, search strategies, and number of resultant records.

Database	Dates of coverage	Search terms	Number of records
CABI digital library	1920–2024	[ab: “transmission”] OR [ab: “introduction”] OR [ab: “prevention”] OR [ab: “mitigation”] OR [ab: “biosecurity”] OR [ab: “clean*”] OR [ab: “disinfect*”] AND [ab: “foot and mouth”] AND [ab: market$] OR [ab: “sale barn$”] OR [ab: “auction market$”] OR [ab: “saleyard$”] OR [ab: “livestock market$”] OR [ab: “stockyard$”] AND NOT [ab: “hand foot and mouth”]	128
Scopus	1800s–2024	TITLE-ABS (“transmission” OR “introduction” OR “prevention” OR “mitigation” OR “biosecurity” OR “clean*” OR “disinfect*”) AND TITLE-ABS (“foot and mouth”) AND TITLE-ABS (market$ OR “sale barn$” OR “auction market$” OR “saleyard$” OR “livestock market$” OR “stockyard$”) AND NOT ABS (“hand foot and mouth”) AND LIMIT-TO (LANGUAGE, “English”)	63
Web of Science*	1637–2024	TS = (“transmission” OR “introduction” OR “prevention” OR “mitigation” OR “biosecurity” OR “clean*” OR “disinfect*”) AND TS = (“foot and mouth”) NOT (“hand foot and mouth”) AND TS = (market$ OR “sale barn$” OR “auction market$” OR “saleyard$” OR “livestock market$” OR “stockyard$”)	237

*Search filter utilized to limit to English language.

### 2.2 Eligibility criteria and selection of sources of evidence

The database search results were exported to Zotero^1^ and duplicate records were removed. The resultant set of records was then uploaded into Covidence^2^ for subsequent screening and data charting steps. Additional duplicate records were identified prior to screening by reviewers and by Covidence’s deduplication tool. All titles, abstracts, and full texts were screened by two reviewers working independently (CH, MS) utilizing a proactively developed screening tool (Appendix 1). Disagreements were resolved by discussion to reach a consensus. Peer reviewed reports were included in the review if they were: written in English, discussed FMD-susceptible livestock species common to the U. S. (cattle, swine, sheep, or goats), and contained information regarding FMD introduction into, transmission within or from an auction market facility, or FMD biosecurity strategies (prevention or mitigation) for auction market facilities. Reports describing international trade or activities conducted at import or export markets were excluded. Reports describing livestock markets which did not operate in a comparable manner to U. S. livestock auction markets were also excluded.

### 2.3 Data items, charting process, and synthesis of results

Data from reports included in the review were charted by two reviewers working independently (CH, MS) using a reviewer-developed data extraction tool in Covidence (see text footnote 2). Disagreements were resolved through discussion to reach a consensus. Key data items were charted at the report level and are summarized in Table 2. For each report citation information, study and population characteristics, and outcomes of interest were charted. Briefly, study characteristics included study design, location (country), and the timeframe during which the study took place as reported by the authors. Population characteristics included a description and number of study units (e.g., samples, farms, model scenarios), and a description of the animal type (species and purpose) with auction market contact. Only animal types which were infected with or susceptible to FMD were charted. Outcomes of interest were categorized as FMD biosecurity risks to or from auction markets, FMD biosecurity strategies for auction markets, and FMD outbreak mitigation strategies involving auction markets. Descriptions and estimates of impact or effectiveness were charted for specific outcomes for each report. Data were exported from Covidence (see text footnote 2) into a Microsoft Excel spreadsheet for synthesis and are presented in either tabular or text format.

**Table 2:** Summary and description of key data items.

Data item	Description
Citation information	A list of all authors, article title, journal name, and publication date (year)
Study characteristics	
Location	The country in which the study was conducted or modeled as reported by the authors
Study design	If specified, reviewers charted the study design as reported by authors. If the study design was not explicitly stated in the report, reviewers categorized the study as one of the following: cross-sectional, case control, outbreak investigation, prevalence study, risk analysis, or simulation or model.
Timeframe	The timeframe during which the study took place or was modeled was charted as reported by the authors.
Population characteristics	
Description and number of study units	A description and number of study units, as appropriate for the study design (e.g., samples, farms, model scenarios), was charted based on author reporting.
Animal type	FMD-susceptible or infected livestock that entered, exited, or passed through an auction market were charted as reported by authors by species (cattle, sheep, goats, swine, buffalo) and purpose if available (beef, dairy).
Outcomes	
Outcomes of interest	Reviewers categorized outcomes of interest into the following categories: FMD biosecurity risks to or from auction markets, FMD biosecurity strategies for auction markets, and FMD outbreak mitigation strategies involving auction markets, and charted descriptions and estimates of impact or effectiveness for specific outcomes as reported by the authors.

## 3 Results

### 3.1 Report selection and characteristics

**Figure 1:**
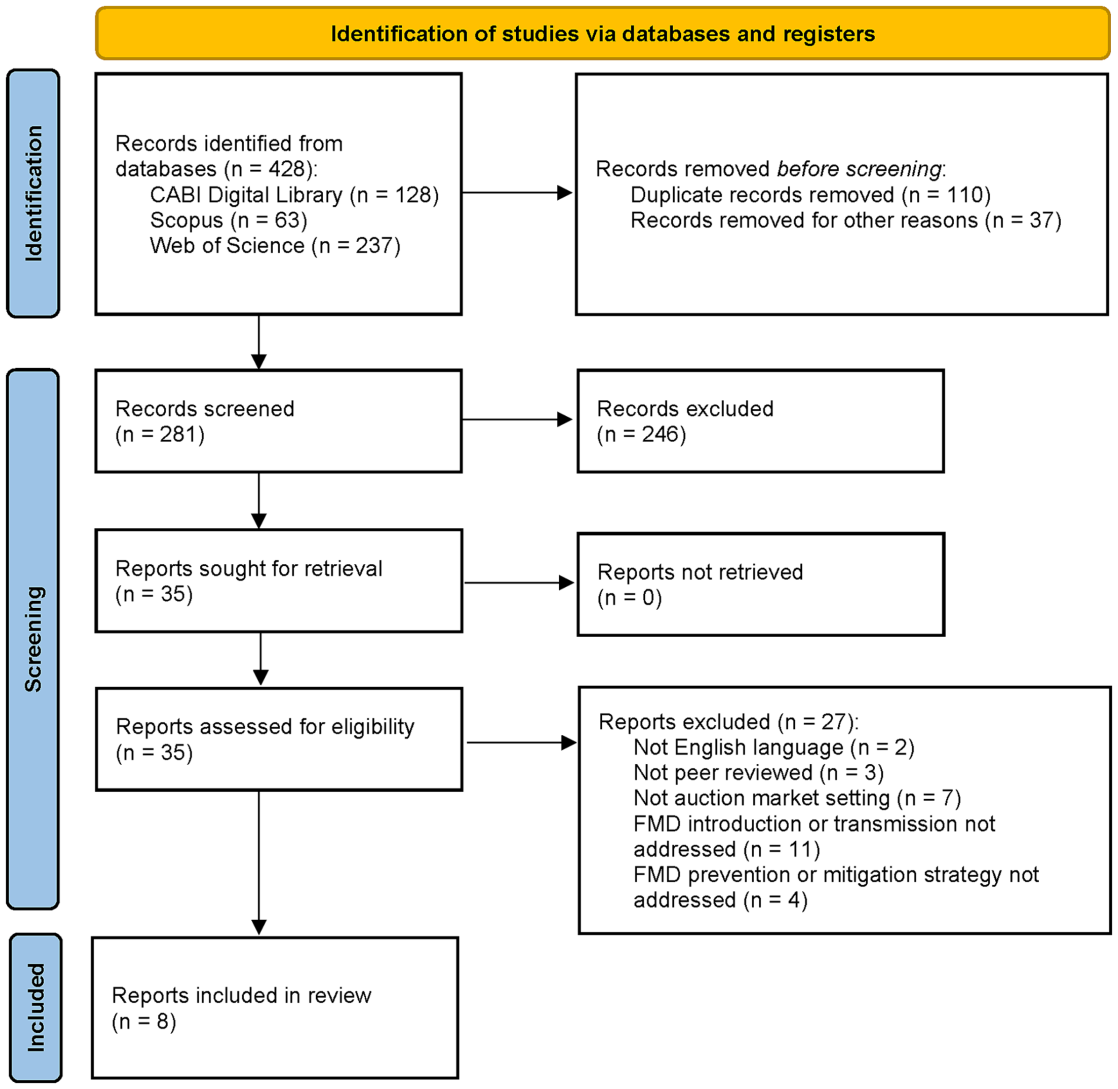
Flow diagram modified from Page et al. of the number of records and reports identified, screened for eligibility utilizing a title and abstract screen followed by a full-text screen, and retained for inclusion in the review (31). Reasons for report exclusion during the full-text screening are detailed.

A total of 428 records were identified from the three database searches. After duplicates were removed, 281 records (title and abstract) were screened, and 35 full text reports were assessed for eligibility with eight reports meeting our criteria for inclusion in the review. Details of the screening process are presented in Figure 1.

The eight reports included in the review were published in five different journals (data not shown) between 2003 and 2023. The reports contained data from seven different countries with data collection timeframes ranging from 2000 to 2018 with one report not specifying a timeframe for the data. Two of the included reports were outbreak investigations, another two described modeling studies, and each of the remaining reports utilized one of the following study designs: case control, cross-sectional, prevalence study, and risk analysis. Six of the reports included data on cattle, three reported data on sheep, two reports each contained data on goats and swine, and one report contained data on mixed cattle and buffalo herds. Six reports included in the review discussed FMD biosecurity risks from auction markets, and two reports discussed FMD outbreak mitigation strategies involving auction markets. No reports describing FMD biosecurity risks to or strategies for auction markets were identified for inclusion in this review. Report characteristics are summarized and presented in Table 3.

**Table 3:** Summary of report characteristics by outcome of interest, study location and timeframe [country, year(s)], animal type with auction market contact, study design, and reference.

Outcome of interest	Country, year	Animal type	Study design	References
Biosecurity risks from auction markets	Cameroon, 2000	Cattle (unspecified type)	Cross-sectional	(18)
	Ecuador, 2002–2003	Beef cattle, Dairy cattle, Cattle (unspecified type), Sheep, Goats, and Swine	Case control	(19)
	Egypt, not reported	Beef cattle and Buffalo	Risk analysis	(20)
	Nepal, 2016–2018	Goats	Prevalence study	(23)
	United Kingdom, 2001	Cattle (unspecified type) and Sheep	Outbreak investigation	(21, 22)
Outbreak mitigation strategies	Australia, 2006–2008	Swine	Simulation/model	(24)
	Thailand, 2013–2014	Cattle (unspecified type)		(25)

### 3.2 Biosecurity risks from auction markets

#### 3.2.1 Quantified estimates of risk from auction markets

Increased risk of introducing FMD into herds was associated with purchasing livestock from markets in three reports. In their cross-sectional study Bronsvoort et al. observed that Cameroon herdsmen who purchased cattle from markets had higher odds of reporting FMD within their herd in the previous year compared to herdsmen who did not purchase cattle from markets (adjusted OR = 2.2, 90% CI = 1.2, 4.1) (18). In their case control study Lindholm et al., observed that case farms (*n* = 39) in Ecuador during the 2002 FMD epidemic were more likely to have purchased cattle from markets compared to control farms (*n* = 78) (adjusted OR = 10.89, 95% CI = 1.82, 65.03) (19). ElAshmawy et al., provided enough information in their risk analysis for the reviewers to calculate an odds ratio and exact 95% confidence interval for the risk of FMD outbreaks in Egyptian feedlots who purchased replacement animals from live animal markets compared to those who purchased replacement from other sources [OR = 4.75 (0.7, 33)] (20). While not significant when utilizing a 95% confidence interval, the risk of FMD outbreaks becomes significant with an 86% confidence interval. Bronsvoort et al., also noted that, in Cameroon, it is not uncommon for cattle to be moved to a market, and if they are not sold, to be returned to the farm of origin (18).

Lindholm et al., also observed that shorter distances from a farm to the nearest livestock market was associated with farms being affected with FMD. Farms that were located less than 10 km or 11–20 km from the nearest livestock market had greater odds of being affected with FMD compared to farms located more than 20 km from the nearest livestock market (<10 km adjusted OR = 39.68, 95% CI = 3.89–402.27; 11–20 km adjusted OR = 12.46, 95% CI = 1.60–96.70) (19).

#### 3.2.2 Descriptions of risk from auction markets

The outbreak investigation reports by Mansley et al., and Thursfield et al., describe how livestock dissemination from auction markets during the 2001 FMD outbreak in the United Kingdom (UK) helped to propagate that outbreak (21, 22). Mansley et al., describe the scope and impact of FMD dissemination from two livestock auction markets; whereas Thrusfield et al., describe the impact of the auction market movements on the region of Dumfries and Galloway (D&G). Both reports focus on the early days of the outbreak, prior to the national livestock movement ban going into effect.

Regarding direct animal movements, Mansley et al., reported that a group of 16 sheep from an infected farm were sold through the Hexham livestock market in three separate groups, two of which resulted in new infected premises. One of the groups of infected sheep (total of 10 sheep) was comingled with 174 additional sheep before being moved to the Longtown auction market where those sheep were sold in 21 different groups, resulting in at least 37 new infected premises due to subsequent movements and sales (21). Thursfield et al., reported that purchasing Longtown market animals was the probable source of infection for 12 premises in D&G, while sharing a common boundary with Longtown market animals was a potential source of infection for another six premises (22).

Thursfield et al. reported that epidemiological links were established between the Longtown auction market and 51 infected premises in D&G (29% of the total infected premises in the region). Epidemiological links were classified as animal, vehicle, and personnel contacts. The authors determined that personnel or vehicle links to Longtown market were the probable source of infection for 16 premises. Early in the outbreak (during the first 3 weeks) animal, vehicle, or personnel contacts with the Longtown market were the source of infection for 8 of 11 clusters of infected premises in D&G (22).

Mansley et al., also suggested that movement of infected sheep through the Longtown market could have resulted in contamination of the market environment, thereby exposing animals to FMD virus which were sold through the market over the next 9 days, until the national livestock movement ban went into effect. The environmental exposure necessitated the tracing of 238 animal movements and could have been the source of infection for 115 premises (21). Thursfield et al., reported that seven dealer premises which became infected due to Longtown market contacts necessitated 1,044 tracings (22).

#### 3.2.3 Auction market environment risk

Colenutt et al., reported that environmental sampling of a goat market in Nepal, where FMD is endemic, yielded detectible FMD RNA in four out of five visits over a two-year period. The authors reported that no veterinary inspections relating to the health of the animals, biosecurity measures, or additional cleaning measures, beyond manure removal, were in place at the market. The study authors were not able to measure infectious virus due to study logistics (23).

### 3.3 Modeling outbreak mitigation strategies involving auction markets

There were no papers reporting empirical data on risk mitigation strategies in auction markets, but two studies modeled potential effects. Hernández-Jover et al., used scenario tree modeling to evaluate the likelihood of Australia’s current disease surveillance system detecting FMD in swine at saleyards that supply either domestic or export markets. Using simulated data of herds and pigs sold to saleyards during a 2-week period, the authors calculated the sensitivity of detecting at least one positive herd assuming a national 1% herd prevalence and a 30% within herd prevalence. Authors reported a median (5 and 95 percentiles) component sensitivity of 0.874 (0.705, 0.936) for domestic saleyards and 0.710 (0.554, 0.840) for export saleyards. The authors also calculated location sensitivities for domestic and export saleyards comparing a 1 or 5% national herd prevalence and a 30, 50, or 80% within herd prevalence. They determined that an increase in national herd prevalence has a greater impact on the likelihood of detecting disease at either type of saleyard than did an increase in within herd prevalence (24).

Wiratsudakul et al., utilized contact modeling to investigate the impact of market closure strategies on an FMD outbreak in Thailand. The authors modeled 15 scenarios: one baseline scenario and four market closure scenarios for each of three markets. The market closure scenarios represented combinations of a one or 2-week lag time in implementing the closure and a one or 2-week closure duration. The difference in lag time did not impact the modeled outbreak sizes. Additionally, closure of a less connected market was not beneficial for decreasing modeled outbreak sizes. However, closure of more highly connected markets reduced the modeled outbreak size by approximately 35.2–41.4% with a one-week closure or by 45.1–47.5% with a 2-week closure (25).

## 4 Discussion

During an FMD outbreak, the ideal situation for an auction market would be to never introduce infected animals into their facility while simultaneously preventing environmental contamination of the market site through all possible routes. Unfortunately, no reports describing FMD biosecurity risks to or strategies for auction markets were identified for inclusion in this review, leaving a gap in available resources to inform preparedness planning and the development of response strategies. The critical need for such resources is illustrated by the five reports included in this review which demonstrate the association between livestock movement through auction markets and FMD introduction risk to herds (18–22). These reports implicate both direct and indirect transmission as well as local spread as pathways which present a risk of FMD herd introduction. The Mansley et al. and Thursfield et al. outbreak investigations illustrate the potential for auction markets to contribute to widespread dissemination of FMD in the absence of biosecurity strategies, inspection requirements, or movement controls, further underscoring their necessity during an outbreak (21, 22). During these outbreak investigations, FMD was disseminated primarily through the movement of infected sheep (21, 22). While sheep do not shed FMD virus quantities as high as other livestock species, particularly swine, they also may not display clinical signs as noticeably as other livestock species (1, 2). The species of livestock infected during an outbreak will affect transmission dynamics of the outbreak, and will likely necessitate tailoring auction market biosecurity and mitigation strategies. For instance, livestock species, like sheep, that shed lower amounts of FMD virus may contribute to less environmental contamination of auction market sites due to less viral shedding; however, since they have less noticeable clinical signs, detection and subsequent outbreak response actions may be delayed. Whereas species that shed higher amounts of the virus and have more obvious clinical signs may contribute to more environmental contamination of premises and local spread via viral aerosolization, but overall time to detection of an outbreak may also be quicker, leading to a more timely response. Additionally, in the U. S., auction markets are utilized as the primary marketing channel for a far smaller volume of swine and sheep compared to other livestock species such as cattle, which will also impact outbreak transmission dynamics (8–12). Regardless of the species infected, practices to prevent entry and rapidly detect clinical animals are necessary. The findings of Wiratsudakul et al. provide further evidence supporting the impact of disease dissemination through markets on the magnitude of outbreaks by demonstrating the reduction in modeled outbreak size from closing highly connected markets (25). This finding was less notable for lowly connected markets, suggesting the need for individual rather than blanket interventions in an outbreak. Bronsvoort et al., ElAshmawy et al., and Lindholm et al. demonstrated the increased risk of FMD herd introduction through the direct movement of livestock purchased from auction markets (18–20). The extensive commingling of animals at auction markets and their subsequent dispersal presents a significant biosecurity risk to both the animals at the market as well as their destination farms. This aspect of the auction markets’ business model highlights the need for effective biosecurity protocols to decrease opportunities for disease transmission and subsequent dissemination.

Surveillance strategies which increase the likelihood of disease detection may also mitigate herd FMD introduction risk from direct movements of livestock purchased from auction markets, such as comprehensive inspections prior to or at the market to detect infected herds as modeled by Hernández-Jover et al. (24). However, these strategies may vary in effectiveness depending on the species infected and virus serotype (26). Moreover, if infected animals are detected at an auction market facility, that facility is likely to face significant challenges regarding best strategies for handling, holding, and potentially disposing of those infected animals. Currently available data identified in this review do not provide specific recommendations for auction markets to implement.

If an auction market facility were to become contaminated either directly by the movement of infected livestock or indirectly through human or vehicle traffic, Colenutt et al. provided evidence of that facility’s ability to serve as a source of viral exposure in both the report included in this review as well as in an additional recently published report (23, 27). However, since both studies were conducted in endemic areas, we cannot distinguish between repeated viral introduction into the auction market and persistence of FMD in the environment between market days. Mielke and Garabed report that FMD virus is capable of surviving in contaminated water sources for 11 to 30 days and in soil for 2 to greater than 30 days (28). Since these reported survival times can exceed the typical interval between sale days for U. S. auction markets, persistent viral load in the market environment is plausible and cleaning and disinfection steps would be necessary to reduce viral exposure and the potential for disease dissemination from auction markets during an outbreak. While cleaning and disinfection guidance is provided by the National Animal Health Emergency Management System (NAHEMS), these processes would be challenging and potentially infeasible for auction markets to repeat regularly (29). Additionally, Mielke and Garabed report a viral survival range of 1–5.5 days for aerosolized FMD virus with the potential for re-suspension of deposited virus particles due to environmental disturbances such as livestock or vehicular movement (28). This capability of the virus poses a risk to newly introduced animals and would necessitate further cleaning and disinfection procedures.

While not all susceptible livestock exposed to a contaminated market site become infected or propagate an outbreak, as illustrated by the Mansley et al. and Thursfield et al. outbreak investigations, the potential viral exposure of the animals, personnel, or other fomites and their subsequent movements do necessitate movement traces during an outbreak (21, 22). In the event of an FMD outbreak in the U. S., the volume of these types of movements originating from a contaminated market site has the potential to obligate personnel resources to a large number of tracing activities, thereby rendering them unavailable for other response activities and placing a strain on all response resources. If available, the implementation of effective biosecurity measures at auction markets could help to mitigate this sort of wastefulness and optimize resource utilization during an outbreak.

Reports included in this scoping review were limited to those published in English which may have eliminated some relevant reports from locations where FMD is endemic. Additionally, only reports from peer-reviewed sources were considered for inclusion in the review, which could have resulted in the exclusion of information relevant to the review questions. However, as there are few countries in which FMD is endemic that also have auction market settings similar to the U. S., the reviewers estimate these limitations to have minimal impact on the results of the review.

In order to reduce the risk of propagating FMD outbreaks due to livestock movement through auction markets while still maintaining some level of business continuity for the auction market sector of U. S. livestock industries, targeted guidance on implementing practical and feasible biosecurity practices and risk mitigation strategies specific to auction market facilities should be developed. Any proposed biosecurity strategies will need to consider and account for the unique business model of U. S. auction markets which result in a high volume of non-terminal livestock movements on a weekly basis. Addressing this current preparedness gap would substantially bolster the U. S. livestock industry’s resiliency in the event of an FMD incursion. Given the clear evidence of widespread dissemination of FMD through auction markets and the limited availability of biosecurity guidance for auction markets, robust implementation of procedures at the farm of origin to prevent movement of infected animals onto the auction market premises along with intensive active observational surveillance at the market is necessary. Farm of origin best practice recommendations for enhanced biosecurity and active observational surveillance are available to U. S producers in the Secure Beef Supply Plan (30).

## Data Availability

The original contributions presented in the study are included in the article/supplementary material, further inquiries can be directed to the corresponding author.

## Footnotes

1 Zotero (Corporation for Digital Scholarship. 2024. Zotero (6.0.36) [Software]. https://www.zotero.org/).

2 Covidence (Covidence systematic review software, Veritas Health Innovation, Melbourne, Australia).

## Appendix 1

Auction Market Biosecurity Scoping Review Screening questions

*Title and Abstract screening.*

Records require a “Yes” answer to all questions below to receive an overall “Yes” classification.

Records that receive a “No” answer to any question below will be assigned a “No” classification.

Records that receive a combination of “Yes” and “Maybe” answers to the questions below will be assigned a “Maybe” classification.

Records that rece ive a “No” classification will be excluded at this step.

Records that receive a “Yes” or a “Maybe” classification will proceed to the full text screening.

1. Are the title and abstract in English?
2. Is the record from a peer-reviewed source?
3. Do the title and abstract indicate that the report contains information regarding FMD introduction into, transmission within or from an auction market facility or information regarding FMD prevention or mitigation strategies for auction market facilities?
4. Do the title and abstract indicate that the report discusses FMD susceptible species common to the U. S. livestock industries?

*Full Text screening*

1. Is the article in English?
  Yes/No ➔ If no, exclude as “Not English language”
2. Is the paper from a peer-reviewed source?
  Yes/No ➔ If no, exclude as “Not peer reviewed”
3. Does the paper contain information regarding FMD introduction into, transmission within or from an auction market facility or information regarding FMD prevention or mitigation strategies for auction market facilities?
  Yes/No ➔ If no, exclude as:
“Wrong setting” if auction market not referenced.
  “Wrong outcomes” if FMD introduction or transmission not addressed.
  “Wrong intervention” if FMD prevention or mitigation strategies not addressed
4. Does the paper discuss FMD susceptible species common to the U. S. Livestock industries?
  Yes/No ➔ If no, exclude as “Wrong population.”
